# Factors associated with changes in students’ self-reported nursing competence after clinical rotations: a quantitative cohort study

**DOI:** 10.1186/s12909-023-04078-7

**Published:** 2023-02-11

**Authors:** H. Ösp Egilsdottir, Lena Günterberg Heyn, Ragnhild Sørum Falk, Espen Andreas Brembo, Kirsten Røland Byermoen, Anne Moen, Hilde Eide

**Affiliations:** 1 Centre for Health and Technology, Faculty of Health and Social Sciences, Institute for Nursing and Health Sciences, University of South-Eastern Norway, Grønland 58, 3045 Drammen, Norway; 2 Oslo Centre for Biostatistics and Epidemiology, Oslo University Hospital, Oslo, Norway; 3 Faculty of Health and Social Sciences, Institute for Nursing and Social Sciences, University of South-Eastern Norway, Grønland 58, 3045 Drammen, Norway; 4 Institute for Health and Society, Faculty of Medicine, University of Oslo, Forskningsveien 2B, 0371 Oslo, Norway

**Keywords:** Nursing students, Clinical placements, Confidence, Nursing care, Physical assessment skills, Clinical skills, Nursing competence, Mobile learning

## Abstract

**Background:**

The quality of nursing care in different healthcare contexts can be associated with the level of available nursing competence. Physical assessment skills are vital in nurses' assessment of patient care needs. However, in nursing education, using physical assessment skills is challenging for nursing students who struggle to apply these skills comprehensively in a clinical rotation. Therefore, this study explores changes in nursing competence, factors associated with changes after clinical rotations, and whether a Suite of Mobile Learning Tools supports changes in confident use of basic physical assessment skills.

**Methods:**

A quantitative cohort study with an explorative pre-and post-test design. During autumn 2019 and spring 2020, 72 s-year nursing students and 99 third-year students participated in the study. The Nurse Professional Competence scale short form was used to investigate students’ self-reported changes in nursing competence, and a study-specific questionnaire was used to investigate students’ confidence concerning performing physical assessments. The students voluntarily used the Suite of Mobile Learning Tools for the learning of physical assessment. Linear regression analysis was used to identify factors associated with changes in nursing competence after clinical rotation. The STROBE guidelines for cohort studies were followed for study reporting.

**Results:**

After the clinical rotation, both student groups reported changes in nursing competence and confidence in performing physical assessment skills, with statistically significant moderate or large changes in all areas. The Suite of Mobile Learning Tools was evaluated as being useful for learning physical assessment. The regression analysis showed that confidence in performing physical assessment skills, the usefulness of the Suite of Mobile Learning Tools, and a higher nursing competence at the start of clinical rotation were positively associated with overall nursing competence.

**Conclusion:**

Basic physical assessment skills are an important component of nursing competence and can be considered one of the pillars of person-centered care, as proposed by the Fundamentals of Care framework. Spaced repetition and access to digital resources are suggested pedagogical approaches to enhance student confidence in the use of assessment skills within academic and clinical contexts.

**Supplementary Information:**

The online version contains supplementary material available at 10.1186/s12909-023-04078-7.

## Background

Highly skilled and competent nurses are increasingly needed in all clinical healthcare contexts due to demographic population projections, emerging new treatment modalities and technologies, task shiftings and new professional responsibilities, and global health challenges [1]. The overall quality of patient care provided can depend on the availability of competent nurses [2, 3]. Undergraduate nursing education, with its combination of theoretical and practical learning objectives and outcomes, intends to provide graduated nurses with the necessary nursing competence to provide safe and high-quality patient care. Nursing competence is a multidimensional and dynamic concept encompassing nurse’s knowledge, understanding, judgment, and cognitive, technical, psychomotor, and interpersonal skills [4, 5]. Systematic and structured health assessment is a core element of nursing competence, crucial for the nurse’s clinical reasoning capacity and ability to provide person-centred holistic care [4, 6].

Performing health assessments includes a collaboration between the nurse and the patient, where the focus is on assessing the health situation and collecting objective information about the patient and subjective information from the patient [6]. Through a systematic and structured approach, a full and comprehensive health assessment entails a) physical assessment, where the nurse uses a range of assessment skills to collect the needed objective information, and b) history taking, where the nurse uses communication skills to inquire subjective information [6]. Subsequently, the nurse collaborates with the patient to identify current care needs, followed by clinical decision-making to determine appropriate nursing interventions. As such, health assessments are vital for the nurse’s scope of practice [7].

Despite the importance of mastering physical assessment skills within health assessment processes, research shows that nurses only use a limited range of their learned physical assessment skills in clinical practice [8, 9]. Moreover, the question of whether too many physical assessment skills are being taught in undergraduate nursing education has been raised [7–9]. Different studies have identified which skills are most frequently used by nurses in clinical practice [9, 10]. Therefore, to ensure “depth” instead of “breadth” of skills, careful consideration was done related to which physical assessment skills to prioritize in the undergraduate nursing education program at our university, termed Basic Physical Assessment Skills (B-PAS) [11]. The examination techniques in B-PAS consist of inspection, palpation, percussion, and auscultation, which are used to systematically collect relevant information. B-PAS is structured after four body systems a) respiratory assessment, b) peripheral circulation and heart assessment, c) abdominal assessment, and d) neurological assessment. These assessments comprise nurses' most frequently used skills [7–10].

To scaffold the nursing students’ (hereafter “students”) learning and training of B-PAS throughout the three-year bachelor’s nursing education, a Progression Model was developed (Supplementary File 1, [11]). The pedagogical assumption “spaced repetition” underpinning the model assumes that applying, training, and repeating the same skills through all three years will enhance students’ confidence in using B-PAS., The effect of practicing the same elements in different contexts leads to increased learning [12]. Perceived confidence, also known as self-efficacy is a person’s belief in their ability to successfully perform a certain task [13] in this case, B-PAS. Self-efficacy can strongly impact learning outcomes when developing more confidence through repeated success when performing certain tasks [13]. This is facilitated through repetitive training in using and performing B-PAS with peers and faculty in the university’s skill lab and clinical rotation. The increasing complexity of the health assessment and use of B-PAS in different contexts throughout the three-year education program is emphasized in the Progression Model by including and highlighting cognitive, clinical, and relational skills [11]. Furthermore, learning, the transfer of knowledge, and students’ successful utilization of specific skills across different contexts is a complex process where contextual factors play a key role [14]. To better support the learning of B-PAS in different contexts, a Suite of Mobile Learning (mLearning) Tools was co-designed with students, enabling training that supports skills acquisition and knowledge transfer processes between educational and clinical contexts [15]. Specific questionnaire items were developed for the current study to map students’ perceived confidence in using B-PAS in clinical rotation and the usefulness of the Suite of mLearning Tools.

### Basic physical assessment skills, Fundamentals of Care, and nursing competence

The Fundamentals of care (FoC) Framework provided a lens for interpretation of students’ self-reported data in the study. The framework highlights the core dimensions of nursing [16] and has also been described by Kitson [17] as a point-of-care nursing theory. The FoC Framework emphasizes factors influencing the delivery of person-centered care in a three-layer model. A prerequisite for person-centered care is establishing a therapeutic relationship between the nurse and the patient (and their family) [16]. The FoC Framework is divided into three main areas: a) the nurse–patient relationship (which within the context of this study represents the student–patient relationship), b) integration of care, and c) contextual factors. The nurse–patient relationship is further underpinned by dynamic processes based on the following five elements: i) establishing trust, ii) being focused on the patient, iii) anticipating the patient’s spoken and unspoken care needs, iv) engaging in knowing the patient through communication, and v) evaluating the provided care with the patient and/or their family [16]. The second area, integration of care, addresses the holistic approach to physical and psychosocial care needs that depend on the relational caregiver’s (i.e., nurse/student) actions. This dimension of the model draws attention to the patient’s fundamental care needs, for example, emotional wellbeing, mobility, and feeling safe. The third area, contextual factors, are factors at a system and policy level that influence the integration of care and the relationship between the student (nurse) and the patient. These factors can include employee resources, the organization of care, the ward culture, and regulations in the healthcare sector [16]. The patient’s essential needs—which the nurse’s assessment should capture—thus depend on the current health situation and in which context the care is or should be provided.

It has been argued that biomedical perspectives have dominated contemporary nursing practice with an increased focus on specific tasks, checklists, and cost-effective organization of care. This can lead to an overly instrumental and technical understanding of nursing care and a devaluation of other key aspects conceptualized as fundamentals of care— with a corresponding risk of losing sight of other core elements of nursing care [16]. For example, the value of the nurse–patient relationship underpinned by person-centered beliefs and values [16]. Our perspective, in conducting the current research is that integrating those perspectives does not exclude one or the other perspectives: both knowledge traditions are essential for nurses to deliver high-quality care and enhance patient safety.

Despite being underpinned by biomedical knowledge (for example, pathophysiology and anatomy), performing health assessments including B-PAS is an important part of the student (nurse)–patient relationship in the FoC Framework. Thus, gathered subjective and objective (through using basic physical assessment skills) information and students’ knowledge about the patient greatly influences their assessment and selection of interventions toward the processes of integrating care and preventing the likelihood of missed care needs. However, the outcomes of health and physical assessments rely heavily on how the relationship and collaboration are between the student and the patient. The level of competence (for example, regarding communication, relational, and clinical skills) that the student brings into the student–patient relationship influences the integration of care [16, 18]. Acquiring skills to communicate, collaborate, and confidently perform B-PAS in a person-centered way can enhance the patient’s feelings of trust, being cared for, and being informed about their health condition. On the other hand, if a student lacks communication skills and confidence in performing B-PAS, this increases the risk of missed care needs and hinders successful integration of care.

Students’ abilities in providing person-centered care have been associated with their level of nursing competence [19]. No survey has been developed to measure or explore the integration of care in the FoC Framework. However, several instruments have been used to assess students’ level of competence: for example, the Nurse Professional Competence scale short form (NPC-SF; 20). The NPC-SF measures self-reported nursing competence related to six competence areas: a) Nursing Care, b) Value-Based Nursing Care, c) Medical and Technical Care, d) Care Pedagogics, e) Documentation and Administration of Nursing Care, and f) Development, Leadership, and Organization of Nursing Care (Supplementary File 2; [20]).

The NPC-SF has been used to measure students’ competence at the point of graduation and nurses’ self-reported competencies shortly after graduation [20, 21]. Research shows that students typically achieve higher scores related to areas of Nursing Care, Value-Based Nursing Care, Medical and Technical care, and Documentation and Administration of Care. The competence areas related to Development, Leadership, and Organization of Nursing care, and Care Pedagogics seem to represent areas where students report lower competence scores [21–23]. However, the role of B-PAS in relation to these six competence areas has not been explored. From an educational perspective, it is essential to continuously identify changes in nursing competence in undergraduate nursing programs [3]. This is regarded as a key strategy that can provide clear recommendations about needed improvements in the curriculum, which can help secure the relevance and quality of the overall competence students achieve by the end of nursing education [3, 20]. To our knowledge, no study has systematically explored the changes in students’ competence after clinical rotations in the different educational years, their perceived confidence in performing B-PAS (the examination techniques), and the usefulness of having access to a Suite of mLearning Tools—or the associations between components.

## The study

### Aims

This study aimed to explore changes in nursing competence, factors associated with changes after clinical rotation, and whether a Suite of mLearning Tools supports changes in confident use of B-PAS. The specific research questions were:

- 1. How do the students evaluate the Suite of mLearning Tools supporting the use of B-PAS in clinical rotation?
- 2. Do the students report changes in confidence in performing B-PAS after one clinical rotation in the second and third educational years?
- 3. Do the students report changes in competence after one clinical rotation in the second and third educational years?
- 4. Which factors are associated with the changes in overall nursing competence?

### Design

This is a quantitative cohort study with an explorative pre-and post-test design. The cohort study design is particularly useful due to exploring the differences between the second- and third-year students [24]. The STROBE guidelines for cohort studies were followed for study reporting (Supplementary File 3).

### Participants

All second-and third-year students at one university campus were invited to participate in the study. The students received oral and written information about the study (regarding the aim, voluntary participation, the content of the Suite of mLearning Tools, and how to withdraw from the study). The oral information was provided in classes before the planned clinical rotation and the written information was posted on the university’s learning management system, Canvas. The same information was also presented at the simulation center when the students participated in the preparation classes before the clinical rotation, together with a presentation of the Suite of mLearning Tools.

### The Suite of mLearning Tools

The Suite of mLearning Tools that was, as reported earlier, co-designed with students in 2019, from all three years in the nursing education program [15], accessible on Canvas by personal computer, tablet, or smartphone and was available only for the students participating in the study. The Suite of mLearning Tools contains a careful selection of tailored digital learning resources aimed to support the learning and application of B-PAS [15]. The participating students could access the Suite of mLearning Tools as much or as little as they preferred during the eight weeks of clinical rotation. Hence, the extent of students’ use of the Suite of mLearning Tools was voluntary. Non-participating students received the ordinary clinical rotation, as described in the Progression model [11], but did not have access to the Suite of mLearning Tools. An overview of the content of the Suite of mLearning Tools can be found in Supplementary File 4 [15].

### Data collection

**Fig. 1:**
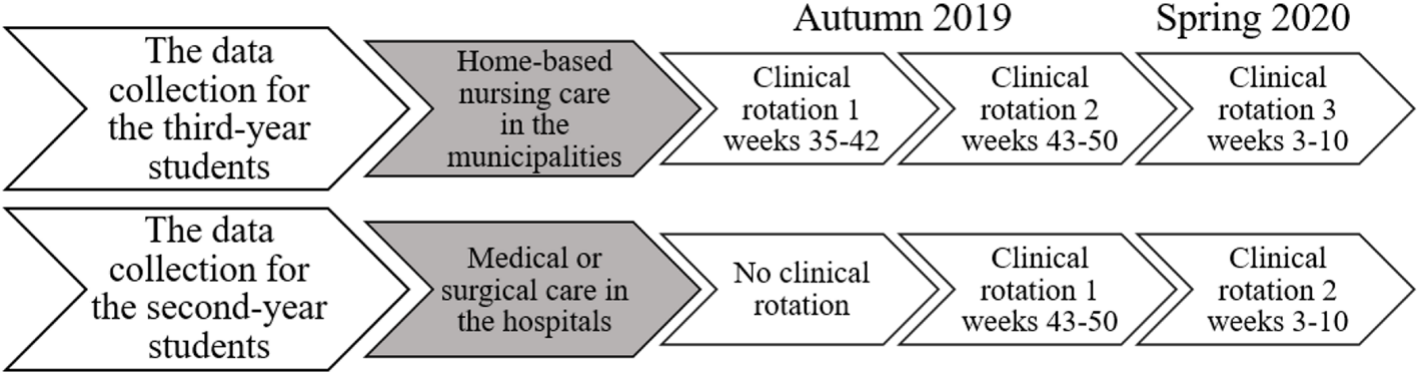
Overview of the clinical rotation periods where the data was collected before and after these clinical rotation

Data collection was carried out in the autumn of 2019 and the spring of 2020 when the second-year students had entered their medical or surgical clinical rotation period in hospitals, and when the third-year students had started their home-based nursing care clinical rotation period in the community healthcare services (Fig. 1). The clinical rotation periods were organized in both the autumn and spring semester and the data were collected before and after one of the clinical rotation periods. The supervision model was the same for all the clinical rotations entailing that the preceptor supervised only one nursing student during the eight weeks of clinical rotation.

**Fig. 2:**
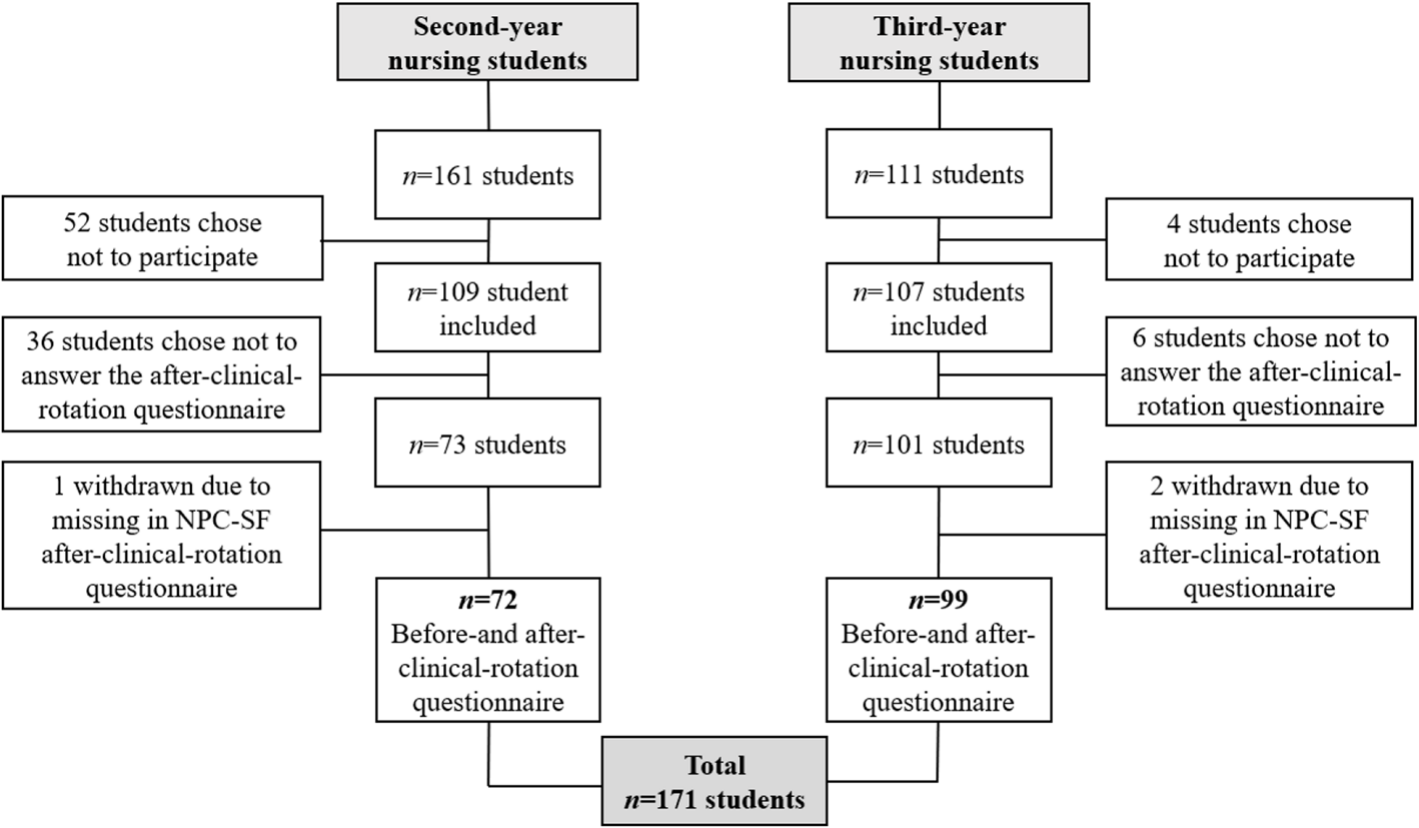
Total number of participating students in the study

The data collection procedure was identical in all the clinical rotation periods. The before-clinical-rotation (pre-test) questionnaire was administered to both student groups at the university campus before the students started their respective clinical rotations. The first author was available for potential questions regarding the questionnaire. The faculty members supervising the students in the clinical rotation collected the after-clinical-rotation (post-test) questionnaire in situ. Both questionnaires were administered via paper and pencil. A total of 109 (of 161, or 68%) second-year students and 107 (of 111, or 96%) third-year students answered the before-clinical-rotation questionnaire (Fig. 2). Among the participating students, 73 (45%) of the second-year students, and 101 (90%) of the third-year students returned the after-clinical-rotation questionnaire.

### The questionnaires

The questionnaire consisted of four sections: a) the Nurse Professional Competence scale short form (NPC-SF), b) items measuring students’ perceived confidence related to performingB-PAS, c) items related to students’ evaluation of the Suite of mLearning Tools, and d) the sample characteristics.

#### The nurse professional competence scale short form (NPC-SF)

The NPC-SF measures self-reported nursing competence through 35 items distributed across 6 competence areas (Supplementary File 3). The 35 items are presented as a question starting with “Do you think you have the ability to…” followed by an example of a competence statement relevant to nursing. One example is “Do you think you have the ability to respectfully communicate with patients, relatives, and staff?” Another example is “Do you think you have the ability to independently perform or participate in examinations and treatments?” The participants were asked to express how much they agreed with each item by using a 7-point Likert scale, where 1 = to a very low degree, 2 = to a relatively low degree, 3 = to some degree, 4 = to neither low nor high degree, 5 = to some degree, 6 = to a relatively high degree, and 7 = to a very high degree. The subscales for the six competence areas were converted into scores between 1 and 100, where 100 indicates the highest possible self-reported competence score [20].

#### Confidence in performing B-PAS

A questionnaire with 13 items was created for this study to map the student’s perceived confidence related to the examination techniques in B-PAS: inspection, palpation, percussion, and auscultation. In the respiratory and abdominal assessments, all four techniques are relevant for use. In the peripheral circulation and the heart assessment, three techniques are used (inspection, palpation, and auscultation), whereas in neurological assessment, only two techniques are relevant (inspection and palpation). The questionnaire items were formulated for the examination techniques within each B-PAS area and in line with Bandura's [13] description of self-efficacy. A 7-point Likert scale ranging from 1 (to a very low degree) to 7 (to a very high degree) was used for all responses. An overall confidence B-PAS score was created as a sum of the 13 items converted into a 1 to 100 score, where 100 indicates the highest possible perceived confidence score.

#### Evaluation of the Suite of mlearning Tools

Eleven items were created to assess the extent to which each of the components of the Suite of mLearning Tools had contributed to the students’ use of the B-PAS. In addition, one item was created to ask the students to evaluate the overall influence of the Suite of mLearning Tools on the use of B-PAS in clinical rotations*.* The same 7-point Likert scale as for the NPC-SF and confidence was used for the responses.

#### Sample characteristics

The nursing students provided background information such as age, gender, educational course, and work experience before enrolling in nursing education and on whether they had work experience in the healthcare sector.

### Ethical considerations

The Norwegian centre for research data (NSD) approved the study (Project No. 674624). According to the national regulations, further approval from a medical ethical committee was unnecessary since the purpose of the study was not to generate new knowledge about health and illness. The institutional research board at the university approved the study. All participants were informed about confidentiality, voluntary participation, the use of the Suite of mLearning Tools, how to withdraw from the study, and how the researchers would store and manage the collected data. After signing the informed consent form, the students were given access to the Suite of mLearning Tools in Canvas. The students were also informed that participation in the study would not influence the formal evaluations they received in the clinical rotation period.

### Data analysis

The statistical package IBM SPSS version 28 was used for data analysis [25]. Descriptive statistics were presented as frequencies with proportions for categorical data and as mean with standard deviation, median, and range for continuous data. The missing data in the NPC-SF were replaced with a group mean within each item, as described in Gardulf et al. [3]. Questionnaires with more than 60% missing data were excluded (*n* = 3). The same procedure was performed with missing items related to mapping students’ confidence. The difference between before and after clinical rotation measurements in the NPC-SF’s six competence areas and the student’s confidence was compared by paired sample *t*-test. If *p* =  < 0.001, Cohen’s *d* was calculated and interpreted as a small (> 0.2), moderate (> 0.5), or large (> 0.8) effect size [26].

#### Construction of the overall NPC-SF score

To further explore changes in students’ competence, a decision was made to construct an overall NPC-SF score, comprising the sum of the 35 items converted into a 1 to 100 score, where 100 indicates the highest possible self-reported competence score. It was conditional that the overall score had a positive relationship with all six competence areas within the NPC-SF instrument. The correlation analysis revealed moderate to strong correlations.Table 1 shows the mean, standard deviation (SD), and Pearson’s correlation coefficient (r) for the NPC-SF six competence areas (CA), the overall NPC-SF score, and the overall B-PAS confidence score after clinical rotation among nurse students (*n* = 171).

**Table 1:** Correlation table for the construction of the overall NPC-SF score

After clinical rotation			CA 1	CA 2	CA 3	CA 4	CA 5	CA 6	Overall NPC-SF score
	*Mean*^*h*^	*SD*	*r*	*r*	*r*	*r*	*r*	*r*	*r*
CA 1^a^	81.8	10.1							
CA 2^b^	88.3	8.5	.580						
CA 3^c^	82.2	9.5	.717	.627					
CA 4^d^	78.4	10.6	.565	.576	.625				
CA 5^e^	83.7	9.0	.687	.629	.786	.581			
CA 6^f^	71.0	12.4	.575	.490	.692	.660	.682		
Overall NPC-SF score	80.9	8.4	.813	.751	.892	.794	.887	.846	
Overall B-PAS^*g*^ confidence score	75.6	12.2	.463	.372	.537	.350	.519	.412	.534

^a^ Nursing Care

^b^ Value-Based Nursing Care

^c^ Medical and Technical Care

^d^ Care Pedagogics

^e^ Documentation and Administration of Nursing Care

^f^ Development, Leadership, and Organization of Nursing Care

^g^ Basic Physical Assessment Skills

^h^ Scores ranged from 1 to 100, where 100 indicates the highest possible self-reported competence/confidence score

Further, we used factor analysis to determine whether an overall NPC-SF was reasonable. The Kaiser–Meyer–Olkin measure of sampling adequacy was 0.91, exceeding the recommended value of 0.6 [27]. Bartlett’s test of Sphericity reached statistical significance, supporting the factorability of the correlation matrix. Kaiser’s criterion and a scree test were examined to determine the number of factors. A one-factor solution was prominent, as the variance explained a decline from 41 to 2% from the first to the second component, and the scree plot showed a clear break after the first component. To aid in the interpretation of the component, a direct oblimin rotation was performed. The component matrix showed a loading above 0.4 for all the items around 1 component. In summary, the calculation of an overall NPC-SF score was suitable, and thus used in the subsequent analyses.

#### Factors associated with the change in overall NPC-SF

A linear regression analysis was performed to evaluate which factors were associated with the change in the student’s nursing competence, with the overall NPC-SF score after clinical rotation as the outcome. The overall B-PAS confidence score and the overall NPC-SF score before clinical rotation, along with the overall usefulness of the Suite of mLearning Tools after clinical rotation, were the variables of interest. In addition, year of education, gender, and age were included as independent variables to adjust for potential confounding factors. The assumption of linearity of the continuous variables was met. No multicollinearity was observed. Results are presented as beta coefficients with 95% confidence intervals (CI).

### Validity and reliability

The NPC-SF is validated and has shown good validity and reliability in earlier studies [20, 21]. The reliability of the NPC-SF has been reported as good to excellent with Cronbach’s alpha values ranging from 0.71 to 0.86 for all factors [20]. In this study, Cronbach’s alpha values ranged between 0.83 and 0.89 for the 6 competence areas measured before the clinical rotation. As shown above, the calculation of an overall NPC-SF score seemed reasonable.

To our knowledge, no validated instrument exists to explore students’ perceived confidence in performing a physical assessment, nor to evaluate the usefulness of the Suite of mLearning Tools. It was, therefore, necessary to create study-specify items for the purpose of this study. The confidence items contain four statements related to each examination technique for example: “I am confident that I can inspect correctly when assessing the respiratory system” and tailored the different focus of B-PAS. The same example for the neurological assessment was worded as: “I am confident that I can inspect correctly when assessing the neurological system”. A total of 13 items were created, four for the respiratory assessment, three for the peripheral circulation assessment and the heart, four for the abdominal assessment, and two for the neurological assessment (Table 4). The 13 items in the overall B-PAS confidence showed good internal consistency with Cronbach’s alpha over 0.9. For the evaluation of the usefulness of the Suite of mLearning Tools eight items were created, seven items evaluated the specific content of the mLearning Tools, and one item evaluated the overall usefulness of the Suite of mLearning Tools (Table 3). Both these sections of the overall questionnaire showed good face validity.

## Results

### The sample

The characteristics of the students participating in this study are presented in Table 2. Most of the students were female (90%) and Norwegian citizens (84%). A majority (74%) had some work experience before starting their nursing education, 40% of the students had 1 to 5 years of work experience in the healthcare sector. A total of 36 (33%) students in the second year and 6 (6%) students in the third year did not respond to the after clinical rotation questionnaire. Non-responder analysis showed that 81% of the students were female and 79% had working experience. The overall B-PAS confidence score and the overall NPC-SF score before clinical rotation were similar to the responses among students who returned both questionnaires (Supplementary File 5).

**Table 2:** Characteristics of the two groups of nursing students

	**Second-year nursing students**	**Third-year nursing students**	**Total *n* = 171 Frequency (%)**
	*n* = 72 **Frequency (%)**	*n* = 99 **Frequency (%)**	
**Gender**			
**Female**	67 (93.1)	87 (87.9)	154 (90.1)
**Male**	3 (4.2)	10 (10.1)	13 (7.6)
**Unspecified**	2 (2.8)	2 (2.0)	4 (2.3)
**Age, years**			
**≤ 20**	32 (44.4)	28 (28.3)	60 (35.0)
**21–25**	22 (30.6)	32 (32.3)	54 (31.6)
**26–30**	6 (8.3)	11 (11.1)	17 (9.9)
**31–35**	6 (8.3)	11 (11.1)	17 (9.9)
**36–40**	3 (4.2)	7 (7.1)	10 (5.8)
**≥ 41**	3 (4.2)	10 (10.1)	13 (7,6)
**Nationality**			
**Norwegian**	68 (94.4)	75 (75.8)	143 (83.6)
**Other**	4 (5.6)	24 (24.2)	28 (16.4)
**Work experience before enrolling in nursing education**			
**No work experience**	19 (26.4)	26 (26.3)	45 (26.3)
**Assistance**	22 (30.6)	28 (28.3)	51 (29.8)
**Nurse assistance**	10 (13.9)	11 (10.1)	21 (12.3)
**From different health** **profession**	10 (13.9)	18 (18.2)	28 (16.4)
**Not from healthcare** **services**	11 (15.3)	15 (15.2)	26 (15.2)
**Years of work experience in the healthcare sector**			
**≤ 1**	33 (45.8)	51 (51.5)	84 (49.1)
**1–5**	31 (43.1)	37 (37.4)	68 (39.9)
**6–10**	5 (6.9)	8 (8.1)	13 (7.6)
**11–15**	3 (4.2)	1 (1.0)	4 (2.3)
**Unspecified**	0	2 (2.0)	2 (1.2)

### The evaluation of the Suite of mLearning Tools at the end of the clinical rotation

The content in the Suite of mLearning Tools was rated higher by the third-year students compared to the second-year students, with a median score ranging from 5.0 to 6.0 and 4.0 to 5.0, respectively (on a 1 to 7 Likert scale; Table 3). The information related to auscultation skills was evaluated highest by both student groups. The third-year students also assessed that the video lectures contributed to increased use of B-PAS. The overall usefulness of the Suite of mLearning Tools was evaluated higher among the third-year students (median 6.0) compared to the second-year students (median 5.0).

**Table 3:** Evaluation of the individual content and overall evaluation of the Suite of mLearning Tools

Individual content in the Suite of mLearning Tools	Second-year nursing students (*n* = 72)	Third-year nursing students (*n* = 99)
	Mean (*SD*) [Median]	Mean (*SD*) [Median]
Instructional videos contributed to the increased use of the B-PAS^a^ in clinical rotation	3.94 (1.64) [4.0]	5.38 (1.27) [5.0]
Video lectures contributed to the increased use of the B-PAS^a^ in clinical rotation	4.06 (1.61) [4.0]	5.42 (1.33) [6.0]
Additional information about auscultation of the lungs and heart contributed to increased use of the B-PAS^a^ in clinical rotation	4.28 (1.62) [5.0]	5.48 (1.28) [6.0]
Virtual simulation contributed to increased use of the B-PAS^a^ in clinical rotation	4.07 (1.64) [4.0]	5.20 (1.55) [5.0]
MOOC^b^ contributed to increased use of the B-PAS^a^ in clinical rotation	3.57 (1.39) [4.0]	4.61 (1.36) [5.0]
Podcasts contributed to the increased use of the B-PAS^a^ in clinical rotation	3.50 (1.37) [4.0]	4.54 (1.40) [5.0]
MCQ^c^ contributed to increased use of the B-PAS^a^ in clinical rotation	3.97 (1.49) [4.0]	5.14 (1.39) [5.0]
Overall use of the Suite of mLearning Tools contributed to increased use of the B-PAS^1^ in clinical rotation	4.39 (1.60) [5.0]	5.73 (1.20) [6.0]

*SD* Standard deviation

^a^Basic Physical Assessment Skills

^b^Massive Open Online Course

^c^Multiple Choice Questions

### Changes in perceived confidence in performing B-PAS

Both the second-and third-year students rated their perceived confidence in performing B-PAS with a mean ranging from 4.0 to 6.1 (on a 1 to 7 Likert scale) and reported similar confidence mean scores at the beginning of the clinical rotation (Table 4). Among the second-year students, only the inspection and palpation related to peripheral circulation assessment reached a small effect size while the percussion related to abdominal assessment reach a moderate effect size. Among third-year students, the changes in perceived confidence reached significantly moderate to large effect sizes within all the B-PAS areas, with the lowest confidence score being related to auscultating the heart. Changes in the overall B-PAS confidence score were statistically significant in both student groups, but only clinically significant among the third-year students (effect size > 0.8). The correlation matrix in Table 1 also shows how the overall B-PAS confidence score correlated with all 6 competence areas and the overall NPC-SF score after clinical rotation.

**Table 4:** Students’ perceived confidence in performing B-PAS

	**Second-year nursing students (*n* = 72)**			**Third-year nursing students (*n* = 99)**		
	**Before clinical rotation**	**After clinical rotation**		**Before clinical rotation**	**After clinical rotation**	
**The specific B-PAS**^**a**^** areas**	Mean (*SD*) [Median]		*p* value^b^ (Cohen’s *d*^c^)	Mean (*SD*) [Median]		*p* value^b^ (Cohen’s *d*^c^)
**The respiratory assessment**						
I am confident that I can inspect correctly when assessing the respiratory system	4.57 (1.22) [5.0]	4.89 (1.22) [5.0]	0.051	4.61 (1.32) [5.0]	5.75 (0.87) [6.0]	< 0.001 (0.93††)
I am confident that I can palpate correctly when assessing the respiratory system	4.32 (1.27) [4.0]	4.82 (1.11) [5.0]	0.002	4.35 (1.37) [5.0]	5.52 (0.91) [6.0]	< 0.001 (1.0)
I am confident that I can percuss correctly when assessing the respiratory system	4.22 (1.25) [4.0]	4.73 (1.06) [5.0]	0.001	4.20 (1.21) [5.0]	5.39 (1.0) [5.0]	< 0.001 (1.2)
I am confident that I can auscultate the lungs correctly when assessing the respiratory system	4.54 (1.18) [5.0]	5.00 (1.10) [5.0]	0.004	4.46 (1.39) [5.0]	5.68 (0.98) [6.0]	< 0.001 (0.99)
**The peripheral circulation assessment and the heart**						
I am confident that I can inspect correctly when assessing the peripheral circulation	4.90 (1.16) [5.0]	5.53 (0.98) [6.0]	< 0.001 (0.62)	5.35 (1.00) [5.0]	6.09 (0.66) [6.0]	< 0.001 (0.78)
I am confident that I can palpate correctly when assessing the peripheral circulation	4.77 (1.28) [5.0]	5.37 (0.99) [5.0]	< 0.001 0.47	5.28 (0.10) [5.0]	5.97 (0.72) [6.0]	< 0.001 (0.67)
I am confident that I can auscultate the heart correctly when assessing the peripheral circulation	4.30 (1.40) [5.0]	4.79 (1.02) [5.0]	0.005	4.44 (1.42) [5.0]	5.27 (1.20) [5.0]	< 0.001 (0.62)
**The abdominal assessment**						
I am confident that I can inspect correctly when assessing the abdominal system	4.70 (1.10) [5.0]	4.89 (1.24) [5.0]	0.187	4.79 (1.20) [5.0]	5.70 (0.96) [6.0]	< 0.001 (0.81)
I am confident that I can auscultate the heart correctly when assessing the abdominal system	4.66 (1.22) [5.0]	4.89 (1.21) [5.0]	0.134	4.96 (1.18) [5.0]	5.84 (0.93) [6.0]	< 0.001 (0.79)
I am confident that I can palpate correctly when assessing the abdominal system	4.43 (1.11) [5.0]	4.85 (1.19) [1.19]	0.004	4.78 (1.21) [5.0]	5.66 (0.97) [6.0]	< 0.001 (0.74)
I am confident that I can percuss correctly when assessing the abdominal system	4.10 (1.18) [4.0]	4.58 (1.15) [5.0]	< 0.001 **(**0.48)	4.53 (1.23) [6.0]	5.40 (1.05) [6.0]	< 0.001 (0.71)
**The neurological assessment**						
I am confident that I can inspect correctly when assessing the neurological system	4.33 (1.07) [5.0]	4.50 (1.26) [5.0]	0.319	4.46 (1.31) [5.0]	5.32 (1.02) [5.0]	< 0.001 (0.64)
I am confident that I can palpate correctly when assessing the neurological system	4.01 (1.19) [4.0]	4.27 (1.26) [4.0]	0.111	4.28 (1.30) [5.0]	5.30 (0.96) [5.0]	< 0.001 (0.73)
**The overall B-PAS**^**a**^** confidence score**^**d**^	63.6 (13.3) [65.9]	69.4 (12.2) [71.4]	< 0.001 (0.40)	66.5 (14.2) [67.0]	80.1 (10.1) [81.3]	< 0.001 (1.2)

^a^ Basic Physical Assessment skills

^b^ *p* value from paired sample t-test, considered statistically significant if < 0.001

^c^ Cohen’s *d* interpreted as moderate effect size if > 0.5 and large effect size if > 0.8

^d^ Values for the overall confidence scores ranged from 1 to 100, where 100 corresponds to the highest level of perceived confidence

### Changes in nursing competence

The difference in the students’ responses before and after one clinical rotation reached statistical significance in all six competence areas (Table 5). The changes were measured as moderate or large in all areas in both student groups, with the largest effect size (> 0.8) being related to Nursing Care, and Medical and Technical Care. The second-year students reported the lowest moderate change (> 0.53) within the area of Development, Leadership, and Organization of Nursing Care, whereas the third-year students reported the lowest moderate change (> 0.72) related to Care Pedagogics. The change in overall NPC-SF score from before to after clinical rotation was statistically and clinically significant in both student groups.

**Table 5:** Nursing students self-reported changes within the six competence areas before and after one clinical rotation

NPC-SF Competence areas		**Second-year students (*n* = 72)**			**Third-year students (*n* = 99)**		
		**Before clinical rotation**	**After clinical rotation**	*p* value^b^ (Cohen’s *d*^c^)	**Before clinical rotation**	**After clinical rotation**	*p* value^b^ (Cohen’s *d*^c^)
	Cronbach’s alpha^a^	Mean (*SD*)			Mean (*SD*)		
Nursing Care	0.84	67.9 (10.93)	77.2 (10.84)	< 0.001 (0.89)	77.5 (9.02)	85.0 (8.05)	< 0.001 (0.82)
Value-Based Nursing Care	0.80	79.4 (10.28)	86.6 (8.65)	< 0.001 (0.64)	83.9 (9.55)	89.6 (8.26)	< 0.001 (0.74)
Medical and Technical Care	0.83	66.6 (10.43)	78.7 (9.83)	< 0.001 (1.0)	76.4 (9.77)	84.7 (8.37)	< 0.001 (0.88)
Care Pedagogics	0.87	64.4 (12.35)	75.2 (10.80)	< 0.001 (0.76)	73.2 (11.60)	80.7 (9.91)	< 0.001 (0.72)
Documentation and Administration of Nursing	0.86	72.7 (10.36)	80.6 (9.40)	< 0.001 (0.77)	78.9 (9.62)	86.0 (8.10)	< 0.001 (0.76)
Development, Leadership, and Organisation of Nursing Care	0.87	57.8 (13.56)	66.1 (12.41)	< 0.001 (0.53)	66.2 (10.97)	74.6 (11.22)	< 0.001 (0.74)
**The overall NPC-SF score**^**d**^	0.96	68.2 (9.29)	77.4 (8.34)	< 0.001 (1.01)	76.0 (8.54)	83.5 (7.46)	< 0.001 (1.02)

*SD* = standard deviation and NPC-SF = Nurse Professional Competence scale Short Form

^a^ Cronbach’s alpha measured after clinical rotation

^b^ *p* value from the paired *t*-test, considered statistically significant if < 0.001

^c^ Cohen’s *d* interpreted as moderate effect size if > 0.5 and large effect size if > 0.8

^d^ Values for the overall NPC-SF score ranged from 1 to 100, where 100 corresponds to the highest level of self-reported competence

### Factors associated with the change in the overall nursing competence

Univariable linear regression analyses showed positive associations between the individual variables and the overall NPC-SF score after clinical rotation (Table 6).

**Table 6:** Univariable and multivariable linear regression analysis of factors associated with the overall NPC-SF score after clinical rotation (*n* = 171)

	**Univariable analysis**			**Multivariable analysis**		
	Coefficient	95% *CI*	*p* value	Coefficient^a^	95% *CI*	*p* value
Overall B-PAS confidence score before clinical rotation^b^	0.20	0.11, 0.29	< 0.001	0.10	0.03, 0.18	0.008
Overall usefulness of the Suite of mLearning Tools^c^	1.94	1.16, 2.71	< 0.001	0.80	0.09, 1.52	0.028
Overall NPC-SF score before clinical rotation^b^	0.52	0.42, 0.63	< 0.001	0.40	0.28, 0.52	< 0.001

*CI* Confidence interval, *B-PAS* Basic physical assessment skills, and *NPC-SF* Nurse professional competence scale short-form

^a^ Multivariable linear regression coefficients adjusted for all covariates including year of education, gender, and age

^b^ Scores range from 1–100 where higher scores indicate higher self-reported confidence or nursing competence

^c^ Scores range from 1–7 where higher scores indicate better evaluation of the Suite of mLearning Tools

Multivariable linear regression analysis, also adjusted for year of education, gender, and age, revealed consistent results. Students reporting a high overall NPC-SF score before clinical rotation had a higher overall NPC-SF score after clinical rotation; for each unit increase in the “before” score, the average increase in the “after” score was 0.4 (95%, *CI* 0.3–0.5). A positive association was also observed between the overall B-PAS confidence score and the overall Suite of mLearning Tools evaluation score (understood as the degree of the usefulness of the Suite of mLearning Tools). Hence, among the students reporting a high overall B-PAS confidence score and high overall usefulness of the Suite of mLearning Tools, the higher the self-reported overall NPC-SF score was after the clinical rotation (Table 6). The adjusted *R*^2^ showed that the regression model explained 42% of the variance in the overall NPC-SF score after clinical rotation.

## Discussion

This study contributes three main findings. First, confidence in performing B-PAS is an important aspect of students’ overall nursing competence, and of all six competence areas defined by NPC-SF. Second, voluntary use of the Suite of mLearning Tools contributed to change in overall nursing competence by supporting increased use of B-PAS. And third, overall nursing competence increased significantly after clinical rotation in the second and third educational years. These findings will be discussed with regard to the FoC Framework and relevant empirical studies.

### Confidence in B-PAS is important for overall nursing competence

This study is unique in its investigation of students’ perceived confidence specifically related to performing all four examination techniques in B-PAS—*inspection*, *palpation*, *percussion*, and *auscultation*—in second and third educational years. Working with B-PAS requires professional knowledge from human bioscience topics like anatomy and pathophysiology, which underpin appropriate clinical reasoning processes [28]. Based on the findings, B-PAS can be considered a pillar of person-centered care, as proposed by the FoCFramework, and an important element in nursing competence. We found moderate to high correlations between all six competence areas of the NPC-SF and overall B-PAS confidence score (Table 1). The multivariable regression analysis also showed a positive association between overall B-PAS confidence score and overall NPC-SF score after clinical rotation. The higher students rated their confidence in performing B-PAS, the higher their overall NPC-SF score. This highlights the importance of focusing on how to support students in becoming a) competent in the different areas of nursing care during their clinical rotation, and b) confident in using B-PAS.

Low student confidence, lack of role models and time, interruptions, and area of specialty in clinical rotations are identified as common barriers to using B-PAS in patient care [8, 9]. These findings correspond to Byermoen et al. [29], who investigated barriers and facilitating factors for students’ use of B-PAS in clinical rotation. Douglas et al. [8] also showed that low student confidence is associated with low utilization of B-PAS in clinical rotation. This indicates that students need to be supported, supervised, and challenged in using B-PAS by preceptors and other members of the clinical rotation. Lack of confidence in performing these assessment skills increases the risk of not being able to adequately identify patient care needs and select appropriate nursing interventions. It can also negatively affect student–patient relationships and hinder the integration of high-quality nursing care. Interestingly, the overall B-PAS confidence score statistically significantly correlated with all NPC-SF competence areas—not just with Medical and Technical Care. Moreover, findings suggest that confidence in performing the B-PAS is regarded as an essential element in Value-Based Nursing Care and Nursing Care. Thus, we argue that using B-PAS systematically in clinical rotations is crucial skills to provide person-centered fundamental care.

The students rated their confidence in performing B-PAS higher after the clinical rotation in the third educational year than students in the second. This may be explained by the natural development of increased confidence during education through more clinical exposure, training with peers, and increased knowledge. However, it is also relevant to examine in which areas of healthcare service the students were placed. The literature highlights that contextual factors could be potential barriers to students’ use of B-PAS [8, 9, 29]. Clinical rotation contexts at our university represent different foci in the two educational years: medical/surgical nursing care in hospitals in the second year and home-based nursing care in the third. Within hospital settings, physical assessments are routinely performed by medical doctors at admission, who are also available for consultation and follow-up in the clinical situation. The second-year students completing their clinical rotation in hospitals reported a small change in overall B-PAS confidence (Cohen’s *d* = 0.4). In home-based nursing care, however, assessments of clinical situations and patient care needs are typically performed more independently by nurses. Third-year students completing clinical rotations in this context reported a large change in overall B-PAS confidence (Cohen’s *d* = 1.2). These findings may indicate that the preceptors in home-based nursing care acknowledge B-PAS as valuable skills in the “toolbox” of nurses’ health assessment and identification of patient care needs. It might therefore be less intimidating for home-based nursing care students to initiatethe use of B-PAS than for students in the hospital setting [29].

The RNs’ skill in performing B-PAS themselves is worth considering. As the introduction of these skills into the curriculum at our university commenced in 2015 [11], many preceptors might still lack confidence needed to successfully integrate B-PAS as a routine in their daily practice. This may arguably limit their supervision around applying and modeling these skills in clinical contexts. Consequently, students might be reluctant to initiate and use B-PAS for fear of disturbing their relationship with their supervisors [8, 9, 29]. We propose that managers should prioritize postgraduate training for their nurses to strengthen their skills to facilitate students’ learning of B-PAS to be confidently practiced and recognized as an essential part of person-centered nursing care. Students would thus benefit from learning and using B-PAS in authentic clinical situations across different healthcare contexts contributing to the growth of confidence and safe learning experiences with peers preceptors, and other health professionals.

### Digital learning resources support the learning of B-PAS

In the multivariable regression analysis, the usefulness of the Suite of mLearning Tools was positively associated with the overall NPC-SF score. This indicates that these digital learning resources supported the students’ development of assessment skills as nursing competence during clinical rotations.. Ewertsson et al. [14] highlighed that learning practical skills requires frequent training. The spaced repetition underpinning the Progression Model introduced by Egilsdottir et al. [11] offers a pedagogical approach that can help students build confidence by organizing supervised training with peers and faculty throughout their nursing education. This pedagogical approach is supported by Ewertsson et al. [14] and Kang [12]. Moreover, access to different digital learning resources—for example, the Suite of mLearning Tools—creates hybrid learning spaces that help minimize barriers to using B-PAS as well as reducing the “theory–practice gap” [15, 29].

Findings show variation in how students in the different educational years evaluated the usefulness of the Suite of mLearning Tools (Table 3). The third-year students considered the digital learning resources useful for supporting B-PAS when in clinical rotation (median score 5.0–6.0); the second-year students were more insecure in their responses (median score 4.0–5.0). This may reflect different uses of the Suite of mLearning Tools and indicate that the second-year students might not have taken as much advantage of the digital learning resources as the third-year students. These differences in students’ responses further suggest that contextual factors may influence students’ application of B-PAS in clinical rotations. Ewertsson et al. [14] highlight the “situated power” nurses have in clinical contexts, which significantly influences how and what students choose to practice when in clinical rotation.

### Changes in nursing competence in different phases during nursing education

Our results show that self-reported competence increased after clinical rotation in second-and third-year students. However, third-year students rated their competence at the *beginning* of their clinical rotation similarly to second-year students’ rating at the *end* of their clinical rotation. This indicates that they continued to experience improvement in their level of competence between the second and third educational years. As in other studies [21–23], students in both educational years rated their competence highest in Value-Based Nursing Care. It has been argued that Value-Based Nursing Care is underpinned by person-centered care values, which has also been associated with increased standards and quality of fundamental care [16]. Interestingly, the students scored high on their Value-Based Nursing Care competence which might indicate that the students had developed qualities in line with person-centered care practices. Two other competence areas where the students in this study assessed themselves as highly competent were Nursing Care and Medical and Technical Care (Cohen’s *d* > 0.8). These competence areas are also components of the FoCFramework suggesting that the characteristics and foci of the learning situations in which the students are engaged during clinical rotations center around the patients and their families. Hence, these results indicate that the students in this study evaluated themselves as highly competent in providing person-centered fundamental care, as suggested by the FoCFramework.

The competence areas within the NPC-SF where the students in both groups assessed themselves as least competent were the Development, Leadership, and Organization of Nursing Care. These results are comparable to other studies [21–23]. Worth noting is that these studies include only students at the point of graduation, not in other educational years, as in the current study. This highlights the need to review the extent to which learning outcomes related to these areas are appropriately covered in nursing education and clinical rotation. Identifying suitable learning situations in clinical practice can also promote learning related to these competence areas.

Some questions in the NPC-SF competence areas can be viewed as the operationalization of recommendations to establish a therapeutic relationship, as proposed by Feo et al. [18]. These questionscan capture students’ application of the Fundamentals of Care in clinical rotations, the student (nurse)–patient relationship, as well as the skills’ integration of care. The NPC-SF can further be used as an outcome measure, as in the current study when students self-assessment demonstrate highly competent within all areas the questionnaire captured.

### Limitations

Study results reports the nursing students’ subjectively experienced changes in nursing competence and confidence. Therefore, limitations of self-reported data must be taken into consideration. Participants may score or rate themselves according to an ideal, or their self-regard, rather than their actual performance or behavior [30]. There is also a possibility that participants rated their competence according to what was expected of them related to being a student in the second or third educational year. Furthermore, the measurement of confidence in B-PAS and the evaluation of the Suite of mLearning Tools must be interpreted with caution since these questionnaires have not been validated. Although the supervision model was the same for all clinical rotation sites, it is possible that the preceptors might have had variable experience of using B-PAS, which could have influenced the student perceived confidence. As perceived confidence in B-PAS is shown to be an important aspect of fundamental care in this study; future studies should validate the questionnaire to establish how well the questionnaire captures actual confidence in and its relation to other competence areas. Due to this, the explorative nature of the study and as the participants represent only two cohorts at one university, the generalizability of the study’s results is limited. Moreover, it would have also been actual to compare individual students’ use of the Suite of mLearning Tools related to their evaluation of the usefulness of the tools. This was not possible in the current study because of the General Data Protection Regulation.

## Conclusion

The findings in this study is that confidence in performing B-PAS is an important component of nursing competence and in fundamental care. Further, the positive association between reported confidence in B-PAS, the usefulness of the Suite of mLearning Tools, and changes in nursing competence suggest that learning opportunities could improve skills, competence, and also confidence. Spaced repetition and access to digital learning resources may offer a way to enhance skills learning and personal growth. The students’ reporting related to different areas of nursing competence measured with the NPC-SF showed adequate levels of fundamental person-centered care practices. In the context of FoC, the students’ confidence and nursing competence can have positive or negative consequences on the student–patient relationship and the integration of care processes. The influences of contextual factors suggest a need for stakeholders from academia, clinical practice, and students to explore together how the acquisition of B-PAS can be integrated better into daily practice. In this way, confidence will be strengthened for nurses and students, emphasizing the benefits of the B-PAS as a component of the assessment “toolbox” for nursing practice.

More research is needed to explore in-depth the association between perceived confidence and competence, and how to stimulate the successful transfer of skills between different learning contexts in nursing education. Furthermore, it would be useful to explore and compare the actual frequency of B-PAS use among the students in both home-based nursing care and medical/surgical clinical rotations.

## Supplementary Information

**Additional file 1:** **Supplementary File 1.** Basic Physical Assesment Skills (B-PAS) in the bachelor nursing program.

**Additional file 2:** **Supplementary file 2.** Overview of the six competence areas in NPC-SF and the main focuses within each area.

**Additional file 3:** **Supplementary file 3.** STROBE Statement—Checklist of items that should be included in reports of cohort studies.

**Additional file 4:** **Supplementary File 4.** Suite of mLearning Tools.

**Additional file 5:** **Supplementary File 5.** Characteristics of the two groups of nursing students that didn’t return the post-questionnaire.

## Data Availability

The quantitative metadata used and analysed in this study are available on request to the corresponding author, H.Ösp Egilsdottir.

## Abbreviations

B-PAS: Basic Physical Assessment Skills
FoC: Fundamentals of Care Framework
NPC-SF: Nurse professional competence scale short form
CI: Confidence intervals
mLearning: Mobile Learning
SD: Standard Deviation
CA: Competence Areas
