# Supplementary figures and images for: Factors associated with changes in students’ self-reported nursing competence after clinical rotations: a quantitative cohort study
Source: BMC Med Educ. 2023 Feb 11;23:107. doi: 10.1186/s12909-023-04078-7 (PMC9922443; doi:10.1186/s12909-023-04078-7)

Supplementary File 1


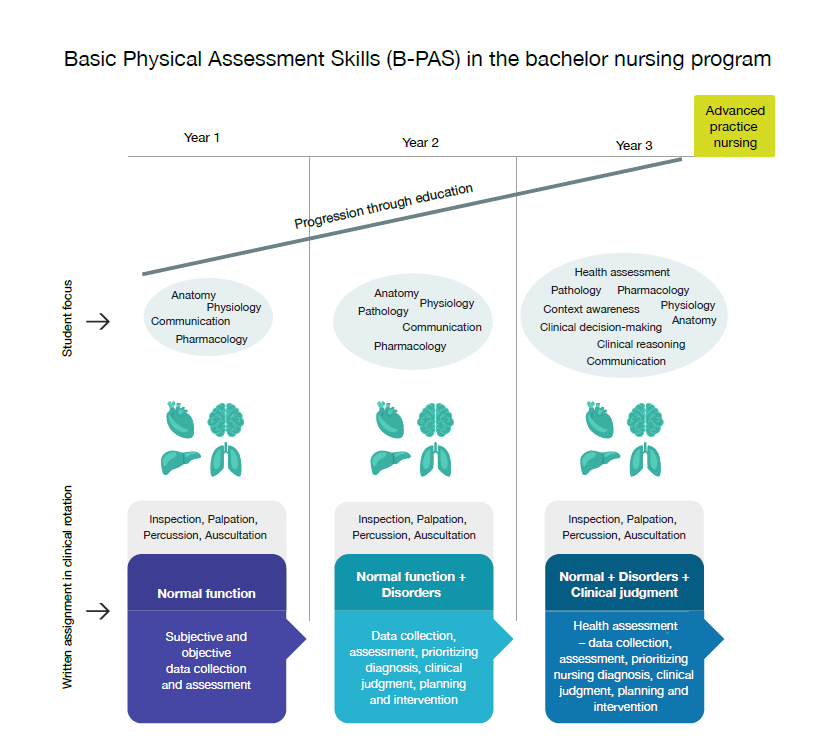

Supplement: Supplementary file 1 — Additional file 1: Supplementary File 1. Basic Physical Assesment Skills (B-PAS) in the bachelor nursing program. [file 12909_2023_4078_MOESM1_ESM.docx]
