# Supplementary material for: Factors associated with changes in students’ self-reported nursing competence after clinical rotations: a quantitative cohort study
Source: BMC Med Educ. 2023 Feb 11;23:107. doi: 10.1186/s12909-023-04078-7 (PMC9922443; doi:10.1186/s12909-023-04078-7)
Supplement: Supplementary file 2 — Additional file 2: Supplementary file 2. Overview of the six competence areas in NPC-SF and the main focuses within each area. [file 12909_2023_4078_MOESM2_ESM.docx]

Supplementary file 2

Overview of the six competence areas in NPC-SF and the main focuses within each area

| The competence areas | | The focus is on mapping competence related to... |
| --- | --- | --- |
| 1 | Nursing Care  (5 items) | collecting and documenting subjective and objective data, assessment, identifying care needs and nursing interventions, and meeting the patients’ physical care needs. |
| 2 | Value-Based Nursing Care  (5 items) | communication skills with patients and their families, a respectful and empathic view of patients’ autonomy and religious beliefs, and capability for teamwork. |
| 3 | Medical and Technical Care  (6 items) | administration of medication, independence in carrying out nursing care, asking questions if unsure, managing medical/technical equipment, and providing care during and after different examinations. |
| 4 | Care Pedagogics  (5 items) | giving support to and including patient and their families in nursing care as well informing and educating them regarding different health conditions. |
| 5 | Documentation and Administration of Nursing Care  (8 items) | documenting patient care, using information systems, patient safety, preventing harm and malpractices, continuous professional development, and coordinating nursing care according to team members’ competence. |
| 6 | Development, Leadership, and Organization of Nursing Care  (6 items) | applying evidence-based practice principles, communicating and collaborating in interprofessional teams, supervising and assessing students’ performance, and managing crises and catastrophic incidence. |
