# Supplementary material for: Factors associated with changes in students’ self-reported nursing competence after clinical rotations: a quantitative cohort study
Source: BMC Med Educ. 2023 Feb 11;23:107. doi: 10.1186/s12909-023-04078-7 (PMC9922443; doi:10.1186/s12909-023-04078-7)
Supplement: Supplementary file 4 — Additional file 4: Supplementary File 4. Suite of mLearning Tools. [file 12909_2023_4078_MOESM4_ESM.docx]

Supplementary File 4

| **Suite of mLearning Tools** | |
| --- | --- |
| **Digital learning resources** | **Content description** |
| 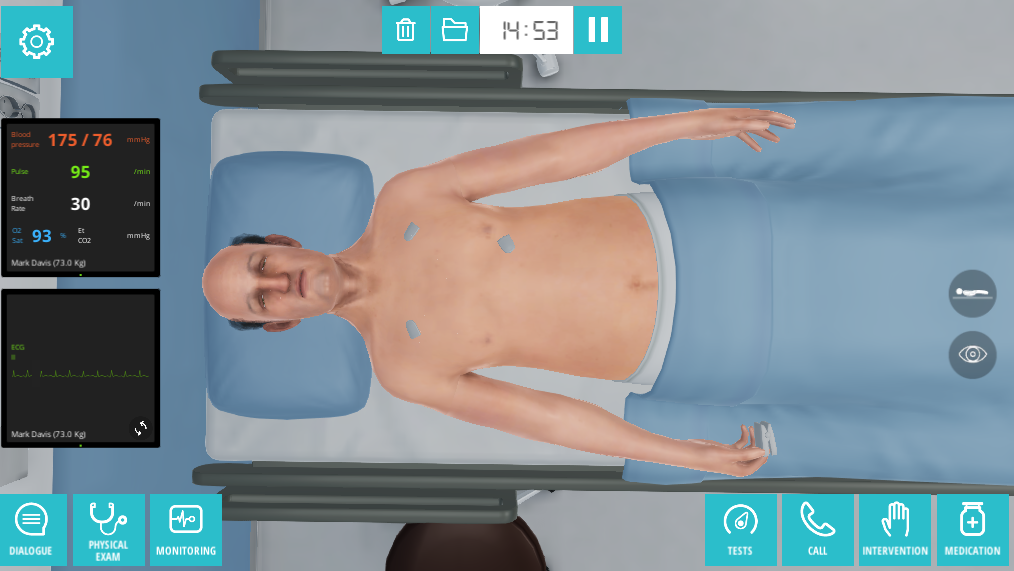 | Short description of the digital simulation program, information about the sessions, and the virtual patient cases on which the student can work. Also included are login details and information about whom to contact if there are problems with the login. |
| 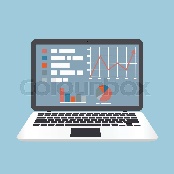 | Brief description of the MOOC^1^ and recommendation for which modules the students should focus on, depending on which educational year they belong to. The students also find details here regarding the login process. |
| 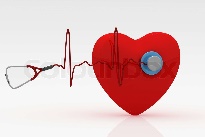 | Detailed information about auscultation skills, divided into two sections: lung sounds and heart sounds. Each section contains links to YouTube videos and audio files with different sounds to which the students may listen. |
| **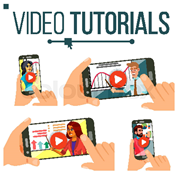** | Five instruction videos with a nurse performing B-PAS^2^ on a patient. Each video has a specific focus: heart and peripheral circulation, the lungs, the abdomen, the neurological assessment, and taking vital signs. The duration of the videos is from 7:37 to 15:51 min. |
| 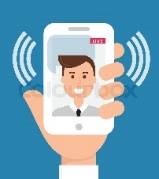 | Four video lectures in which each video has a specific focus: heart and peripheral circulation, the lungs, the abdomen, and the neurological assessment. The duration of the videos is from 13:13 to 32:55 min. |
| **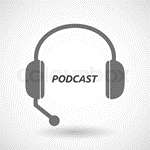** | 1) Two nurses (faculty members) talk about the origin of the physical assessment in nursing education, and the differences between performing B-PAS as a registered nurse (RN) or as a nurse practitioner.  2) A conversation between the faculty members and two newly graduated RNs, focused on working with B-PAS^2^ throughout the three-year nursing program, and how they work with B-PAS^2^ as new RNs. |
| **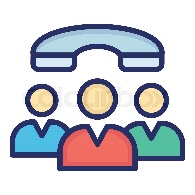** | Brief information about structuring professional communication about data gathered through mapping the patient health condition by using different communication tools, and how to use these tools during clinical rotation. |
| 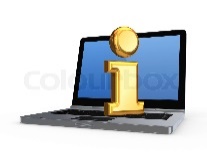 | Information about structuring professional documentation of the information gathered through mapping the patient health condition by using B-PAS^2^ in the different documentation systems used in clinical practice. |
| **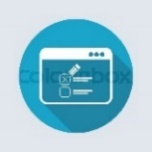** | MCQ^3^ aiming to support students’ knowledge, to repeat and refresh bioscience knowledge, and to identify knowledge gaps. |
| **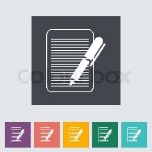** | Description of a written assignment targeting reflection on the use of B-PAS^2^. Feedback is given by fellow students (a peer review for the second- and third-year students), in which all parties are anonymous. |
| **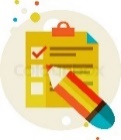** | Checklists summarizing the elements of every focus in B-PAS^2^ (e.g., respiratory system and neurology). Can also be used when students use B-PAS^2^ in clinical rotation. |

^1^: Massive Open Online Course

^2^: Basic Physical Assessment Skills

^3^: Multiple Choice Questions
