# Supplementary material for: Factors associated with changes in students’ self-reported nursing competence after clinical rotations: a quantitative cohort study
Source: BMC Med Educ. 2023 Feb 11;23:107. doi: 10.1186/s12909-023-04078-7 (PMC9922443; doi:10.1186/s12909-023-04078-7)
Supplement: Supplementary file 5 — Additional file 5: Supplementary File 5. Characteristics of the two groups of nursing students that didn’t return the post-questionnaire. [file 12909_2023_4078_MOESM5_ESM.docx]

|  | Second-year nursing students | Third-year nursing students | Total  Frequency (%) |
| --- | --- | --- | --- |
|  | *n*=36  **Frequency (%)** | *n*=6  **Frequency (%)** |  |
| Gender | | | |
| Female | 30 (83.3) | 4 (67) | 34 (81.0) |
| Male | 4 (11.1) | 2 (33) | 6 (14.3) |
| Unspecified | 2 (5.6) |  | 2 (4.8) |
| Age, years | | | |
| ≤20 |  |  |  |
| 21–25 | 21 (58.3) | 1 (16.7)) | 22 (42.3) |
| 26–30 | 8 (22.3) | 3 (50.0) | 11 (26.3) |
| 31–35 | 3 (8.4) | 1 (16.7) | 4 (9.5) |
| 36–40 | 2 (5.6) | 1 (16.7) | 3 (7.2) |
| ≥41 | 2 (5.6) |  | 2 (4.8) |
| Nationality | | | |
| Norwegian | 31 (86.2) | 3 (50) | 34 (81.0) |
| Other | 5 (14) | 3 (50) | 8 (19.2) |
| Work experience before enrolling in nursing education | | | |
| No work experience | 8 (22.2) | 1 (16.7) | 9 (21.4) |
| Assistance | 12 (33.3) | 1 (16.7) | 13 (31.0) |
| Nurse assistance | 6 (16.7) | 1 (16.7) | 7 (16.7) |
| From different health  profession | 7 (19.4) | 3 (50.0) | 10 (23.8) |
| Not from healthcare  services | 3 (8.3) |  | 3 (7.1) |
| Years of work experience from the healthcare sector | | | |
| ≤1 | 19 (52.8) | 1 (16.7) | 20 (47.6) |
| 1–5 | 15 (41.6) | 4 (66.7) | 19 (45.2) |
| 6–10 | 1 (2.8) | 1 (16.7) | 2 (4.8) |
| 11–15 | 1 (2.8) |  | 1 (2.4) |
| Unspecified |  |  |  |
| Overall scores | Mean (*SD*) [*n*] | Mean (*SD*) [*n*] |  |
| Confidence | 64.7 (13.2) [35] | 70.5 (15.0) [6] |  |
| NPC-SF | 69.7 (9.1) [33] | 73.1 (8.4) [6] |  |

Supplementary File 5

Characteristics of the two groups of nursing students that didn’t return the post-questionnaire
